# Common musculoskeletal impairments in postpartum runners: an international Delphi study

**DOI:** 10.1186/s40945-020-00090-y

**Published:** 2020-10-26

**Authors:** Shefali M. Christopher, Alessandra N. Garcia, Suzanne J. Snodgrass, Chad Cook

**Affiliations:** 1 Department of Physical Therapy Education, Elon University, Campus box 2085, Elon, NC 27244 Australia; 2 Discipline of Physiotherapy, School of health Sciences, The University of Newcastle, Callaghan, NSW 2308 Australia; 3 College of Pharmacy & Health Sciences, Doctor of Physical Therapy Program, Campbell University, Lillington, NC 27506 USA; 4 Division of Physical Therapy, Department of Orthopaedic Surgery, Duke University, 2200 W.Main St, Durham, NC 27705 USA

**Keywords:** Postpartum, Running, pain, Injury

## Abstract

**Background:**

Postpartum runners report musculoskeletal pain with running. Because of inadequate research, little is known about the origin and pain-related classification. Through expert consensus, this study is the first attempt to understand the musculoskeletal impairments that these runners present with. The objective of this survey was to gather expert consensus on characteristics of reported impairments in postpartum runners that have musculoskeletal pain.

**Methods:**

A web-based Delphi survey was conducted and was composed of five categories: strength, range of motion, alignment and flexibility impairments, as well as risk factors for pain in postpartum runners.

**Results:**

A total of 117 experts were invited. Forty-five experts completed round I and forty-one completed rounds II and III. The strength impairments that reached consensus were abdominal, hip and pelvic floor muscle weakness. The range of motion impairments that reached consensus were hip extension restriction, anterior pelvic tilt and general hypermobility. The alignment impairments that reached consensus were a Trendelenburg sign, dynamic knee valgus, lumbar lordosis, over-pronation and thoracic kyphosis. The flexibility impairments that reached consensus were abdominal wall laxity, and tightness in hip flexors, lumbar extensors, iliotibial band and hamstrings. The risk factors for pain in postpartum runners were muscular imbalance, poor lumbopelvic control, too much too soon, life stressors, pain during pregnancy and pelvic floor trauma.

**Conclusion:**

This study presents a framework for clinicians to understand pain in postpartum runners and that can be investigated in future cohort studies.

**Level of evidence:**

5

## Introduction

In 2019, USA running reported that 17.6 million people registered for road races with 61% of those registered identifying as female [1]. Of those women 49% were between the ages of 25-44 years, prime childbearing age [1]. The Center for Disease and Control and Prevention reported 29 years as the mean age of women at first childbirth and therefore one can argue that many of the women running may be of childbearing age [2]. A recent survey of female runners reported that 90% of recreation runners exercised regularly during pregnancy, with 72% who ran at any point during pregnancy, and 38% who ran during the third trimester [3]. For those that did not continue to run, reasons such as feeling poorly or uncomfortable, advice from doctor, concern for miscarriage and to gain and maintain weight were noted [4]. After childbirth, one survey reported approximately 50% of competitive participants returned to running at six weeks postpartum and one survey investigating competitive runners reported return to running as early as four weeks [3, 4].

Musculoskeletal pain and dysfunction is prevalent in postpartum runners, lumbopelvic pain in most commonly reported [3]. Among women who returned to running, 35% reported postpartum musculoskeletal pain upon returning to running, with 91% of pain complaints related to the lower back, pelvis and/or hips (lumbopelvic) [3]. Lumbopelvic pain is common during pregnancy and postpartum periods and is reported to affect 50% of pregnant women [4, 5]. This pain has been reported to decrease 1–3 months postpartum [6, 7] for most; however, it can become chronic in up to 7% of women [8]. Lumbopelvic postpartum pain is not well understood. Many risk factors have been hypothesized to be the cause of the pain. Research reporting on pain characteristics, patterns and associations with risk factors is lacking.

Women are running after having a baby and nearly all runners surveyed complained of pain upon returning to running [3]. During the postpartum period women are recovering from several pregnancy related changes such as increased weight gain [5], hormonal changes such as joint and connective tissue laxity, postural changes such as increased lumbar lordosis, flattening of feet [6], transient osteoporosis [7, 8] as well as after effects of the birthing process such as tearing of the pelvic floor muscles or recovering from c-section surgery [9].. First onset lumbopelvic pain has also been reported in postpartum women that did not have pain during pregnancy, due to risk factors related to delivery and maternal demographics [10]. Despite reports of musculoskeletal pain in the postpartum runners, conditions involving musculoskeletal pain are both poorly studied and lack specific measurement tools. To our knowledge there are no studies that have explored characteristics of pain in postpartum runners. In addition, the few existing exercise guidelines for the postpartum running population have been generated primarily from non-postpartum athlete studies [11].

When incomplete evidence exists to assist decision-making, expert opinion is often used in absentia [12–14]. A Delphi technique is a commonly used tool for “decision making and forecasting in a variety of studies” that organizes expert opinion [15]. This technique surveys a group of experts in a designated field to answer a list of sequential questions designed to determine a consensus from the group on a particular topic [16–18]. Consensus methods often help with research that is directed at problem solving, determining priorities, or generating ideas [18]. The purpose of the study was to perform a Delphi survey to gather expert consensus on common characteristics of reported musculoskeletal impairments in postpartum runners with pain, as well as generate expert ideas on common risk factors for pain in postpartum runners.

## Methods

### Study design

This study was a three-round web-based Delphi survey design involving a respondent group and a workgroup [19, 20]. Informed consent was obtained and subjects’ rights were protected.

### Subjects

The respondent group consisted of content expert volunteers, operationally defined as physical therapists or physiotherapists who were first and or last author of a peer-reviewed publication on female running evaluation and treatment and or postpartum evaluation and treatment, or a presenter at either a national or international conference on the topic. Experts were identified through PubMed searches, conference abstracts, and peer review. The authors were invited via email. Unlike surveys, the sample size of Delphi surveys does not depend on statistical power, but on the dynamics of the expert group arriving at consensus [21]. This Delphi aimed for a large sample to reflect all types of clinicians and researchers who interact with postpartum runners.

The workgroup included investigators who were experienced in mixed-methods research, including Delphi investigations. They summarized the data from round one, thematically coded the data and redesigned the follow-up survey instrument [22]. The workgroup as a whole had a minimum of 10 years’ clinical experience and six years’ clinical research experience in orthopedic physical therapy. The lead author was a board-certified sports physical therapist and athletic trainer with over seven years’ experience treating runners. The other investigators have been involved in clinical research for six to 20 years, including one author (XX) who has had first or senior authorship on six Delphi analyses. (reference that would identify author).

### Procedure

The survey consisted of three rounds of questionnaires. Invitations to round I of this study were distributed via Qualtrics email, a survey software which allows collecting and analyzing research data. The email provided a web address link to the consent form and survey. Invitations to rounds II and III survey links were sent to all respondents from round I. Each round was live for 3–4 weeks with weekly reminders.

### Instrument

#### Round I of Delphi

The instrument used in the first round included demographic questions, professional questions and six open-ended questions related to impairments in postpartum runners with musculoskeletal pain. We did not define the location of pain, as we wanted to collect comprehensive information on postpartum runners with pain. We defined postpartum runners as “any female participating in running within two years of giving birth to a baby.” After defining postpartum, the respondents reported the most common strength impairments observed in postpartum runners in the first open-ended question [23]. The following four open-ended impairment-based questions queried topics involving range of motion (ROM), [23] alignment, [23] flexibility, [23] and most common risk factors for pain in postpartum runners. A sixth open-ended question allowed additional comments on the clinical presentation of postpartum runners (Additional file 1) [23].

#### Round II of Delphi

From the qualitative analysis of responses from round I, thematic coding was performed (SMC, ANG, CC). The questions in the second round were a list of impairments for each of the strength, ROM, alignment, flexibility, risk factors, and categories constructed from the thematic coding from round I of the survey. The purpose of round II was to allow all the respondents to review the responses from round I for clarification and correction of terminology, and to identify the most important impairments related to each of the categories in the survey. The respondents used a 4-point Likert scale that ranged from strongly disagree to strongly agree to score the impairments and their level of agreement that the impairment was related to the category included (Additional file 2).

#### Round III of Delphi

**Fig. 1:**
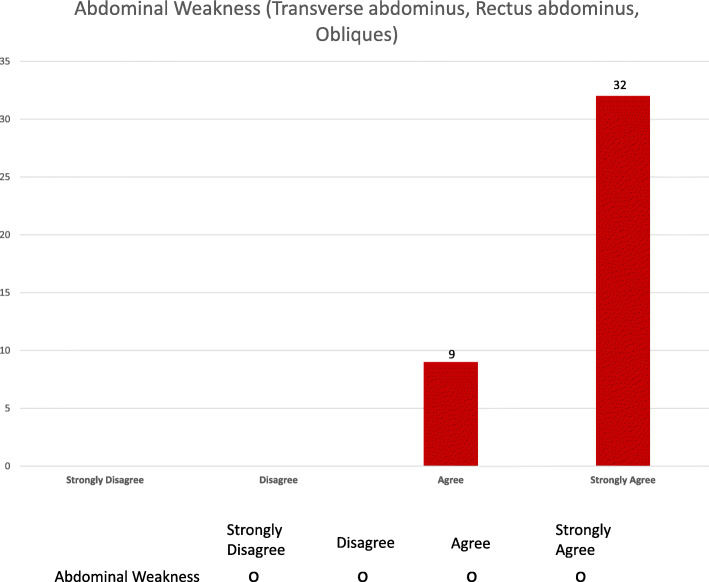
Screenshot of round III survey question using impairment list from round II with additional graphs. X-axis: Likert scale, Y-axis 41 respondents

In round III, the survey instrument was built using the same impairments list and rating scale used in round II with additional graphs demonstrating the descriptive statistical score outcome for each category and impairment. The respondents were asked to re-score each impairment after viewing round II results. Figure 1 corresponds to a screenshot of one round III survey question for the strength category.

### Data analysis

The survey instrument was built on Qualtrics survey software (version XM, Provo, Utah). After each round, the data were downloaded from Qualtrics into an excel spreadsheet for analysis [22]. The impairments in each category that the respondents decided did not relate to the postpartum runner were tallied as strongly disagree or disagree and represented the “Consensus not related” category. The impairments in each category that the respondents decided did relate to the postpartum runner were tallied as strongly agree or agree and represented the “Consensus related” category. A consensus was established if ≥ 75% of the respondents indicated an item as “Consensus not related” or if ≥75% indicated an item as “Consensus related” [24]. In cases where the tally was < 75%, consensus was not established and a decision “Consensus not met” was made [12].

After establishing consensus, the impairments were ranked by composite score using the following formula: Composite score = (n1x 0) + (n2x1) + (n3x2) + (n4x4), where n was the number of respondents, and 1 was “strongly disagree”, 2 was “disagree”, 3 was “agree” and 4 was “strongly agree.” The design of a Delphi survey enables expert respondents to rank composite scores without feedback (Round II) and with graphic feedback (Round III) from other experts, and thus some changes were expected between rounds. Wilcoxon matched pairs signed rank was used to determine the meaningful difference between the scores of round II and III using a *p*-value of < 0.05 [25]. Statistical analyses were conducted using SAS, version 9.4 (SAS institute, Cary, NC).

## Results

### Round I and respondents characteristics

**Fig. 2:**
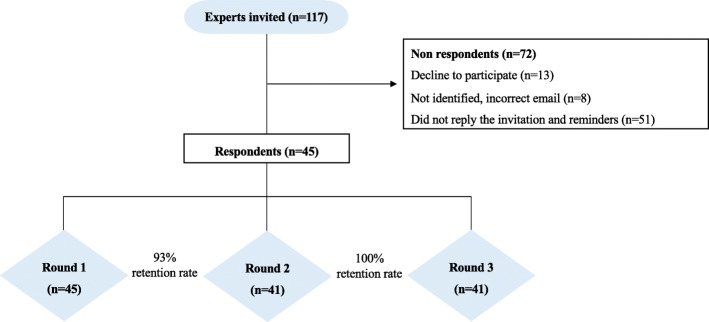
Flow recruitment and study respondents

From March 2018 to June 2018, we contacted 117 content experts from female running or postpartum women’s health content areas. Eight respondents had incorrect email addresses; leaving 109 eligible experts. Thirteen experts declined to participate, noting they were not experts regarding this specific population. Fifty-one participants did not reply to invitations nor reminders. Forty-five participants (41%) completed the consent form and responded to the first round (Fig. 2). Thirty-three respondents were female (73.33%) and twelve were male (26.67%). Four experts (8.89%) resided outside the USA and five (91.11%) in the USA.

These respondents reported 0 to > 20 years of physical therapist practice experience with the largest group (40%) at > 20 years of practice experience. The respondents had 0 to > 20 years of research experience with the largest group (37%) with up to 5 years of research experience. Ten participants (22.22%) had advanced certifications in women’s health and 21 (46.67%) had advanced certifications in orthopedics or sports (Table 1).

**Table 1:** Respondents characteristics in Delphi round I (*n* = 45)

Variables	Number (percentage)
**Demographic variables**	
**Age**	
20–30	5 (11.11)
30–40	16 (35.55)
40–50	14 (31.11)
50–60	7 (15.55)
> 60	3 (6.66)
**Gender**	
Female	33 (73.33)
Male	12 (26.67)
**Country**	
USA	(88.89)
Other	(11)
**Professional variables**	
**Years of clinical practice**	
0–5	7 (15.56)
5–10	10 (22.22)
10–15	4 (8.89)
15–20	6 (13.33)
> 20	18 (40)
**Years of research practice**	
None	4 (8.89)
0–5	17 (37.78)
5–10	9 (20)
10–15	6 (13.33)
15–20	6 (13.33)
> 20	3 (6.67)
**Advanced certifications**	
APTA board specialty (OCS, SCS, WCS)	27 (60)
− WCS	8 (29.62)
− OCS/SCS	19 (70.37)
PhD, EdD, PhDC	9 (20)
Other (i.e., CSCS, ATC, MS, FAAOMPT, CAPP)	19 (42.22)

Experts were physical therapists or physiotherapists who were first and or last author of a peer-reviewed publication on female running evaluation and treatment and or postpartum evaluation and treatment, or a presenter at either a national or international conference on the topic. WCS- Women’s health certified specialist physical therapist, OCS- Orthopaedic certified specialist physical therapist, SCS- Sports certified specialist physical therapist, PhD- Doctor of philosophy, EdD – Doctorate in Education, PhDC- Doctor of philosophy candidate, CSCS- certified strength and conditioning specialist, ATC- athletic training certified, MS- Master of science, FAAOMPT- Fellow of the American academy of orthopedic manual physical therapists, CAPP- certificate of achievement in pelvic physical therapy

### Rounds II and III

Four respondents did not complete the survey from round II despite weekly reminders; 41 of the 45 respondents participated in round II (93% retention rate between rounds I and II, Fig. 2). Forty-one respondents completed round III (100% retention rate between rounds II and III, Fig. 2). A detailed description of total consensus (%) per impairment category for rounds II and III is reported in Table 2.

**Table 2:** Final impairments in Delphi round III for reaching consensus as common musculoskeletal impairments in postpartum runners

Musculoskeletal Impairments	Consensus (%) Round II	Composite Score Round III	Consensus (%) Round III
**Strength**			
Abdominal weakness	100	160	100
Hip abductor weakness	100	154	100
Hip extensor weakness	95.12	142	100
Pelvic floor weakness	95.12	156	97.56
Hip rotator weakness	90.24	136	97.56
**Range of Motion**			
Hip extension restriction	82.93	131	95.12
Excessive counter nutation (anterior pelvic tilt)	90.24	123	92.68
Generally hypermobile, no restriction	68.29	129	90.24
Thoracic extension restriction	75.61	115	78.05
Hip internal rotation restriction	68.29	116	75.61
**Flexibility**			
Tight hip flexors	85.37	150	100
Laxity in abdominal wall	87.80	144	95.12
Tight lumbar extensors	68.29	118	80.49
Tight hamstrings	65.85	115	75.61
Tight iliotibial band	70.73	113	75.61
**Alignment**			
Trendelenburg sign	85.37	129	95.12
Dynamic knee valgus	80.49	124	92.68
Increased lumbar lordosis	87.80	124	92.68
Over pronation	70.73	114	80.49
Thoracic kyphosis	70.73	114	75.61
**Risk Factors**			
Muscular imbalance	100	156	100
Poor lumbopelvic control	100	154	100
Hip weakness	100	152	100
Too much, too soon	95.12	139	100
Trauma to pelvic floor	90.24	138	100
Hip pain	87.80	133	100
Increased life stressors	90.24	133	100
Decreased exercise tolerance	78.05	131	100
Pain during pregnancy	85.37	131	100
Lumbopelvic muscle weakness	97.56	153	97.56
Altered running mechanics	100	143	97.56
Chronic pain history	87.80	140	97.56
Global laxity	78.05	139	97.56
Pelvic floor pain	92.68	137	97.56
Lumbopelvic instability	92.68	137	97.56
Chronic fatigue	80.49	131	97.56
Hip extensor muscle activation	75.61	131	97.56
History of running injury	85.37	141	95.12
Poor sleep quality	82.93	131	95.12
Caretaking posture	73.17	130	92.68
Labor duration	73.17	122	92.68
Increased body mass index (BMI)	78.05	124	90.24

### Impairment categories (see Table 2)

#### Strength

Five strength impairments were ranked as “Consensus related” in postpartum runners in round III. One impairment was ranked as “Consensus not related” and five impairments were ranked “Consensus not met” in round III. The item that was most related to strength impairment was abdominal weakness. Hip abductor (gluteus maximus, medius, minimus) hip extensor weakness were ranked second, followed by pelvic floor weakness and hip rotator weakness. Pectoralis major or minor weakness was ranked as the impairments least related to strength in the postpartum runner.

#### Range of motion

Five ROM impairments were ranked as “Consensus related” in postpartum runners in round III. Seven impairments were ranked as “Consensus not related” and five ROM impairments were ranked “Consensus not met” in round III. The item most related to ROM impairment was hip extension restriction, followed by anterior pelvic tilt, general hypomobility and no restrictions, thoracic extension restriction, and hip internal rotation restriction. Thoracic flexion restriction was the item most not related, followed by knee extension restriction, shoulder flexion restriction, thoracic side flexion restriction, hip flexion restriction, lumbar side flexion restriction, and lumbar flexion restriction.

#### Alignment

Five alignment impairments were ranked as “Consensus related” in postpartum women in round III. One impairment was ranked as “Consensus not related” and 10 items were ranked “Consensus not met” in round III. The item that was most related to alignment impairments in postpartum runners was the Trendelenburg sign. Dynamic knee valgus and increased lumbar lordosis ranked second, followed by overpronation, and thoracic kyphosis. The impairment least related to alignment impairments in postpartum women was posterior pelvic tilt.

#### Flexibility

Five impairments were ranked as “Consensus related” with flexibility impairments in postpartum runners in round III. Seven impairments were ranked as “Consensus not met” in round III. Tight hip flexors were ranked as the top impairment associated with flexibility impairments followed by laxity in abdominal wall, tight lumbar extensors, hamstrings, and iliotibial band.

#### Risk factors

Twenty-three items were ranked as “Consensus related” as risk factors for injury in postpartum runners in round III. Five items were ranked “Undecided” in round III. Muscle imbalance was most related to risk factors for pain in postpartum runners followed by poor lumbopelvic control, hip weakness, too much too soon, trauma to the pelvic floor, hip pain, increased life stressors, decreased exercise tolerance, and pain with pregnancy (all 100% consensus-related).

### Differences between rounds II and III (Table 3)

A meaningful difference was measured between rounds II and III responses (Table 3) [20]. The impairments with significant difference when comparing composite score of rounds II and III (*p* value < 0.05 on Wilcoxon sign rank test) were lumbar extensor weakness and scapular stabilizer weakness in the strength category, thoracic rotation, lumbar extension, and shoulder flexion restriction in the ROM category, tight hip flexors in the flexibility category, anterior pelvic tilt in the alignment category, and runner body type, lumbopelvic instability, diastasis recti, increased Q angle, too much too soon, trauma to the pelvic floor, altered running mechanics, and global laxity in the risk factor category.

**Table 3:**
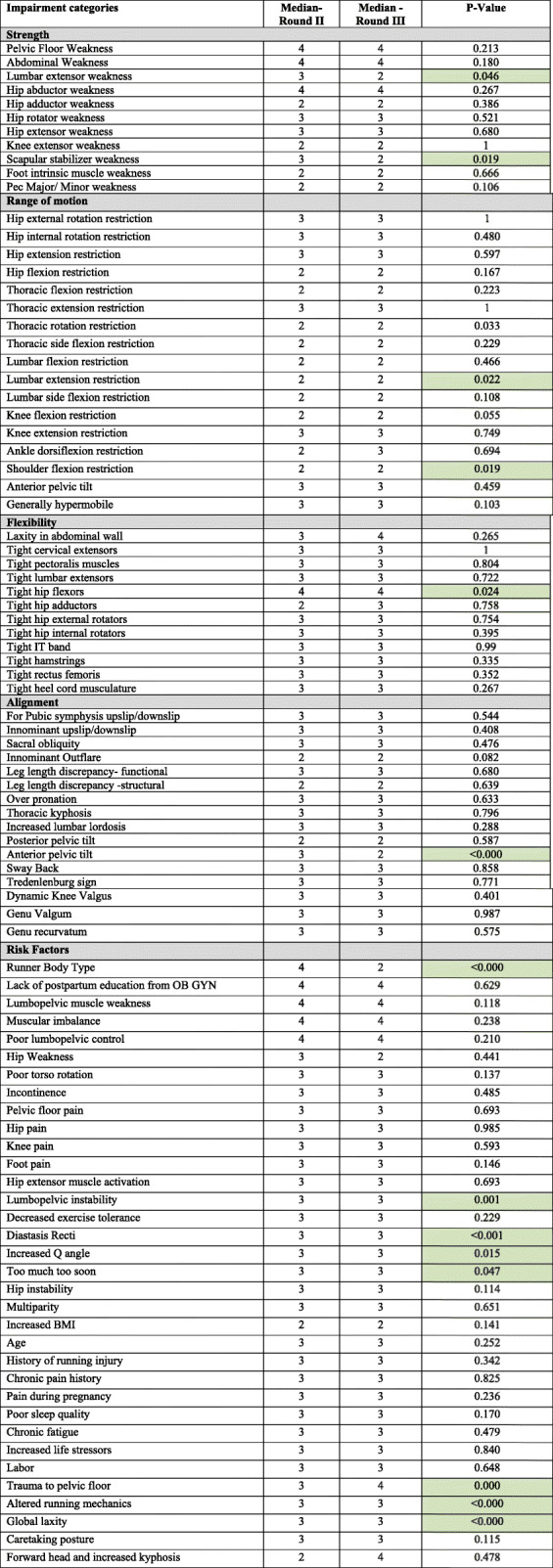
Wilcoxon matched pairs signed rank was used to determine the meaningful difference between rounds using a p-value of < 0.05

Characteristics statistically significant are highlighted in green. Scores on likert scale 1 = strongly disagree, 2 = disagree, 3 = agree and 4 = strongly agree

## Discussion

The purpose of this Delphi study was to identify impairments that contribute to pain with running in the postpartum population. The initial Delphi consisted of forty-one qualified members of the respondent group who finished all rounds who contributed to the results. The study showed a good response rate (100%). Our findings suggest that all impairments meeting the criteria of 75% consensus are potential contributors to pain in the postpartum running population.

Strength impairments that were commonly observed in postpartum runners and reached consensus as impairments were abdominal weakness, hip weakness (rotator, extensor and abductors), and pelvic floor weakness. These factors have only been studied in a limited capacity in the postpartum running population. Two peer reviewed case studies investigating postpartum runners [26, 27] reported the findings of abdominal and hip weakness in the postpartum runners. A recent survey [3] reported 19% of postpartum runners had stress urinary incontinence up to two years postpartum and 27% up to 10 years postpartum, supporting pelvic floor weakness as a strength impairment. Although abdominal, [28–31] pelvic floor, [32] and hip weakness [33] have been documented independently in either the postpartum or running population, high quality evidence from prospective design studies is lacking. While none of these findings are novel in the individual populations, our findings suggest that return to running evaluations in the postpartum population may benefit from core, pelvic floor, and hip strength testing [34–37].

Although laxity is present during pregnancy and postpartum states [38] some flexibility and ROM impairment restrictions that met consensus had conflicting evidence on literature review. Experts reported that tight hip flexors and a limited hip extension ROM were both a flexibility and ROM impairment in postpartum runners. Evidence for this was conflicted, as these impairments were present in one postpartum runner case study [27]; however not present in another [26]. Hip flexor tightness (and lumbar extensor tightness) has been hypothesized to result from postural changes that may take place during pregnancy [39]. Hip flexor stretches are commonly used in treatment programs for pregnancy-related pelvic girdle pain [39, 40]. Tight hip flexors have also been noted in runners compared to non-runners [41]. It could be possible that postpartum runners compensate for laxity with other structures to provide stability [42]. Thus, this Delphi suggests that clinicians should evaluate postpartum runners for these impairments and apply clinical judgement to decide whether the hip requires more motion or the lumbopelvic region requires more strengthening for stability.

The following alignment impairments met consensus for postpartum runners and had conflicting reports in the literature: Trendelenburg sign, dynamic knee valgus, increased lumbar lordosis, overpronation and thoracic kyphosis. In case studies investigating postpartum runners, knee valgus and Trendelenburg sign were supported during functional testing (single leg squat and lunge) in one case study, [26] and increased lumbar lordosis and thoracic kyphosis were noted in one case study [27]. Changes in multiplanar knee laxity have been documented during pregnancy and up to five months postpartum [43]. Pronation has also been documented as a change through pregnancy and postpartum due to laxity and weight gain, and may result in lasting changes in foot structure [44]. Trendelenburg alignment is the result of hip weakness and has been well-studied in cross-sectional studies investigating running injury risk [45–48] and pain [49–51]. Importantly, the association is unclear in prospective studies [23]. As a whole, these findings are supported in the postpartum or running literature. Future studies need further exploration in postpartum runners.

The most common risk factors for postpartum running that were not included in other categories were hip pain, decreased exercise tolerance, pain during pregnancy, too much too soon, life stressors, and pelvic floor trauma. These risks were also studied in either postpartum or running populations. The findings from this category offer a unique perspective in recommendations for returning to running. Pain pre-pregnancy and during pregnancy has been associated with pain in postpartum [52–55]. One of the case studies evaluating pain in the postpartum runner reported a history of pain [27]. Both hip pain and pain during pregnancy could be related to a history of previous injury that has been seen to be a risk factor for future injury in runners [56–60]. Future studies should investigate these factors in pregnant runners as they may also be barriers running during pregnancy and return to running postpartum.

Fatigue, the decreased capacity for activity (either physical or mental due to an imbalance of resources needed to perform an activity), [61] has been reported as the most common problem in the postpartum period, affecting 63.8% of new mothers [62]. Lack of sleep, stress, anxiety and breastfeeding difficulties have all been associated as risk factors for postpartum fatigue [63]. Fatigue has also been studied in the running population as a risk factor for injury [57], [64]. “Too much too soon” has also been studied for its relationship with injury with endurance athletes [65] as high spikes in acute training load have been associated with injury [66]. New mothers may be eager to return to former levels of activity and due to limited peer reviewed guidelines and recommendations, may return to aggressively. A survey of postpartum runners reported nearly 50% of survey participants returned to running at six weeks, sooner than most muscle and fascia healing timelines [3, 11]. Trauma to the pelvic floor was also reported as one of the most risk factors for pain in postpartum runners. During childbirth there can be significant injury to the pelvic floor that may lead to significant problems such as incontinence and prolapse [11]. Muscular imbalance was also reported as a risk factor for pain in postpartum runners. Although studies have not reported on the evaluation of this imbalance, studies focusing on individualized treatment using stabilization exercises have shown higher quality of life, lower disability and lower pain intensity [37]. These Delphi survey findings highlight that postpartum runners may need a team of providers such as a lactation consultant, psychologist, physical therapist, and running coach may assist in reducing pain and injury and that clinicians should include questions related to these risks while evaluating the postpartum runner.

### Limitations

Delphi methodology starts by asking open ended questions followed by voting on the most common answers. This did not allow for us to further understand the expert’s definitions for some of the impairments such as anterior pelvic tilt or hip weakness or risk factor pelvic floor trauma, nor their method of evaluating these impairments. When investigating alignment impairments in postpartum runners, experts ranked anterior pelvic tilt in the “consensus not met” category in round III. An anterior pelvic tilt has been seen to be present as a response to pregnancy and fetal development [67–69]. There is conflicting data in both pregnancy and postpartum [70, 71]. Experts were unable to reach consensus, potentially due to the lack of a clinical reference standard of measurement and conflicting reports of this alignment impairment.

## Conclusion

Postpartum runners report pain with running, yet evidence-based cohort research is lacking about the musculoskeletal impairments and risk factors in postpartum runners with pain. Delphi studies collect and analyze expert information and are often the first step to designing future cohort studies. This Delphi study recorded and analyzed the opinions of physical therapy experts in women’s health and running to provide clinicians with a comprehensive list of possible impairments to more effectively evaluate and treat the postpartum runner in pain. In addition to providing information for clinicians which was previously lacking, researchers will now have a framework with which to design future cohort studies.

## Supplementary information

**Additional file 1.** Delphi Survey instrument used in the first round.

**Additional file 2.** Delphi Survey instrument used in the second round.

**Additional file 3.** Items that were “Consensus not related” or “Consensus not met”.

## Data Availability

All relevant raw data, will be freely available to any scientist wishing to use them for non-commercial purposes.
