# Supplementary material for: Common musculoskeletal impairments in postpartum runners: an international Delphi study
Source: Arch Physiother. 2020 Oct 26;10:19. doi: 10.1186/s40945-020-00090-y (PMC7586674; doi:10.1186/s40945-020-00090-y)
Supplement: Supplementary file 1 — Additional file 1. Delphi Survey instrument used in the first round. [file 40945_2020_90_MOESM1_ESM.docx]

**Appendix A:**

Delphi Survey instrument used in the first round:

For the purpose of this survey, we define postpartum runner as any female participating in running within 2 years of giving birth to a baby.

Q1 What are the most common strength impairments observed in postpartum runners?

Q2 What are the most common range of motion impairments observed in postpartum runners?

Q3 What are the most common alignment impairments observed in postpartum runner?

Q4 What are the most common flexibility impairments observed in postpartum runners?

Q5 What do you believe are the most common risk factors for pain in postpartum runners?

Q6 Do you have any further comments or thoughts on the clinical presentation of postpartum runners?

Demographics:

Q1 What is your age? o <20 o 20-30 o 30-40 o 40-50 o 50-60 o >60

Q2 What gender do you identify with? o Female o Male o Other

Q3 How many years have you been in clinical practice?

o None o 0-5 o 5-10 o 10-15 o 15-20 o 20 or more

Q4 How many years have you been in research?

o None o 0-5 o 5-10 o 10-15 o 15-20 o 20 or more

Q5 Please list any certifications other than physical therapist/ Physiotherapist. Eg. Doctor of physical therapy, Women's health certified specialist.
