# Supplementary figures and images for: Common musculoskeletal impairments in postpartum runners: an international Delphi study
Source: Arch Physiother. 2020 Oct 26;10:19. doi: 10.1186/s40945-020-00090-y (PMC7586674; doi:10.1186/s40945-020-00090-y)

**Appendix B:**

Delphi Survey instrument used in the second round:


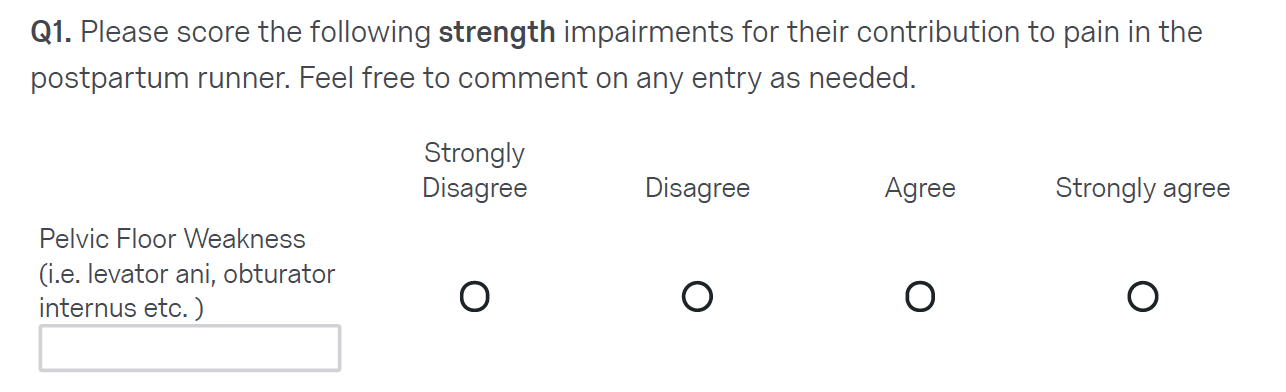

Supplement: Supplementary file 2 — Additional file 2. Delphi Survey instrument used in the second round. [file 40945_2020_90_MOESM2_ESM.docx]
