# Supplementary material for: Common musculoskeletal impairments in postpartum runners: an international Delphi study
Source: Arch Physiother. 2020 Oct 26;10:19. doi: 10.1186/s40945-020-00090-y (PMC7586674; doi:10.1186/s40945-020-00090-y)
Supplement: Supplementary file 3 — Additional file 3. Items that were “Consensus not related” or “Consensus not met”. [file 40945_2020_90_MOESM3_ESM.docx]

**Appendix C**: Items that were “Consensus not related” or “Consensus not met”.

|  | | | | |  |
| --- | --- | --- | --- | --- | --- |
| **Descriptors** | Consensus Status | Round II (%) | Round III  (Composite score) | Round III (%) |  |
| **Strength** | | | | |  |
| Pectoralis Major/Minor Weakness | CNR | 87.8 | 69 | 100 |  |
| Lumbar extensor muscle weakness | CNM | 56.1 | 101 | 43.9 |  |
| Scapular stabilizer weakness | CNM | 53.66 | 100 | 41.46 |  |
| Foot intrinsic muscle weakness | CNM | 56.1 | 96 | 41.46 |  |
| Knee extensor weakness | CNM | 58.54 | 98 | 39.02 |  |
| Hip adductor weakness (adductor longus, brevis, magnus) | CNM | 56.1 | 94 | 29.27 |  |
| **Range of Motion** | | | | |  |
| Thoracic flexion restriction | CNR | 82.93 | 77 | 100 |  |
| Knee flexion restriction | CNR | 75.61 | 82 | 92.68 |  |
| Shoulder flexion restriction | CNR | 78.05 | 79 | 92.68 |  |
| Knee extension restriction | CNR | 68.29 | 91 | 90.24 |  |
| Thoracic side flexion restriction | CNR | 65.85 | 84 | 90.24 |  |
| Hip Flexion restriction | CNR | 75.61 | 83 | 90.24 |  |
| Lumbar side flexion restriction | CNR | 65.85 | 86 | 87.8 |  |
| Lumbar flexion restriction | CNM | 51.22 | 73 | 60.98 |  |
| Hip external rotation restriction | CNM | 53.66 | 105 | 53.66 |  |
| **Alignment** | | | | |  |
| Posterior pelvic tilt | CNR | 75.61 | 86 | 80.49 |  |
| Genu valgum | CNM | 58.54 | 114 | 73.17 |  |
| Leg length discrepancy (F) | CNM | 60.98 | 112 | 73.17 |  |
| Sway back | CNM | 60.98 | 111 | 68.29 |  |
| Innominant outflare | CNM | 56.10 | 96 | 68.29 |  |
| Sacral obliquity | CNM | 60.98 | 110 | 63.41 |  |
| Pubic symphysis upslip/downslip | CNM | 65.58 | 110 | 63.41 |  |
| Innominant upslip/ downslip | CNM | 58.54 | 109 | 60.98 |  |
| Genu recurvatum | CNM | 56.10 | 106 | 60.98 |  |
| Leg length discrepancy (S) | CNM | 60.98 | 94 | 60.97 |  |
| Anterior pelvic tilt | CNM | 90.24 | 99 | 51.22 |  |
| **Flexibility** | | | | |  |
| Tight heel cord musculature | CNM | 56.10 | 116 | 70.73 |  |
| Tight hip external rotators | CNM | 63.41 | 114 | 70.73 |  |
| Tight pectoralis muscles | CNM | 60.98 | 109 | 65.85 |  |
| Tight hip internal rotators | CNM | 56.10 | 110 | 63.41 |  |
| Tight rectus femoris | CNM | 65.85 | 113 | 63.41 |  |
| Tight cervical extensors | CNM | 56.10 | 107 | 58.54 |  |
| Tight hip adductors | CNM | 53.66 | 103 | 51.22 |  |
| **Risk Factors** | | | | |  |
| Foot pain | CNM | 60.98 | 115 | 73.17 |  |
| Age | CNM | 51.22 | 98 | 63.4 |  |
| Increased Q angle | CNM | 58.54 | 102 | 60.98 |  |
| Runner body type | CNM | 53.66 | 100 | 57.5 |  |
| Poor torso rotation | CNM | 51.22 | 102 | 56.10 |  |

CNR- Consensus not reached; CNM: Consensus not met
